# Engineered Ripening-Specific Accumulation of Polyamines Spermidine and Spermine in Tomato Fruit Upregulates Clustered C/D Box snoRNA Gene Transcripts in Concert with Ribosomal RNA Biogenesis in the Red Ripe Fruit

**DOI:** 10.3390/plants9121710

**Published:** 2020-12-04

**Authors:** Vijaya Shukla, Tahira Fatima, Ravinder K. Goyal, Avtar K. Handa, Autar K. Mattoo

**Affiliations:** 1 Sustainable Agricultural Systems Laboratory, The Henry A. Wallace Beltsville Agricultural Research Center, Agriculture Research Service, U.S. Department of Agriculture, Beltsville, MD 20705-2350, USA; wishukla@gmail.com (V.S.); ravinder.goyal@canada.ca (R.K.G.); 2 Department of Horticulture and Landscape Architecture, Purdue University, 625 Agriculture Mall Drive, West Lafayette, IN 47907-2010, USA; fatimat@purdue.edu (T.F.); ahanda@purdue.edu (A.K.H.); 3 Agriculture and Agri-Food Canada, Pulse and Legume Physiology, Lacombe Research and Development Centre, 6000 C & E Trail, Lacombe, AB T4L 1W1, Canada

**Keywords:** small nucleolar RNA, polyamines, spermidine, ribosomal proteins, RNA sequencing, RNA polymerases, translational proteins, tomato, snoRNA clusters

## Abstract

Ripening of tomato fruit leads, in general, to a sequential decrease in the endogenous levels of polyamines spermidine (SPD) and spermine (SPM), while the trend for the diamine putrescine (PUT) levels is generally an initial decrease, followed by a substantial increase, and thereafter reaching high levels at the red ripe fruit stage. However, genetic engineering fruit-specific expression of heterologous yeast S-adenosylmethionine (SAM) decarboxylase in tomato has been found to result in a high accumulation of SPD and SPM at the cost of PUT. This system enabled a genetic approach to determine the impact of increased endogenous levels of biogenic amines SPD and SPM in tomato (579HO transgenic line) and on the biogenesis, transcription, processing, and stability of ribosomal RNA (rRNA) genes in tomato fruit as compared with the non-transgenic 556AZ line. One major biogenetic process regulating transcription and processing of pre-mRNA complexes in the nucleus involves small nucleolar RNAs (snoRNAs). To determine the effect of high levels of SPD and SPM on these latter processes, we cloned, sequenced, and identified a box C/D snoRNA cluster in tomato, namely, *SlSnoR12*, *SlU24a*, *Slz44a*, and *Slz132b*. Similar to this snoRNA cluster housed on chromosome (Chr.) 6, two other noncoding C/D box genes, *SlsnoR12.2* and *SlU24b,* with a 94% identity to those on Chr. 6 were found located on Chr. 3. We also found that other snoRNAs divisible into snoRNA subclusters A and B, separated by a uridine rich spacer, were decorated with other C/D box snoRNAs, namely, J10.3, Z131a/b, J10.1, and Z44a, followed by z132a, J11.3, z132b, U24, Z20, U24a, and J11. Several of these, for example, *SlZ44a*, *Slz132b,* and *SlU24a* share conserved sequences similar to those in Arabidopsis and rice. RNAseq analysis of high SPD/SPM transgenic tomatoes (579HO line) showed significant enrichment of RNA polymerases, ribosomal, and translational protein genes at the breaker+8 ripening stage as compared with the 556AZ control. Thus, these results indicate that SPD/SPM regulates snoRNA and rRNA expression directly or indirectly, in turn, affecting protein synthesis, metabolism, and other cellular activities in a positive manner.

## 1. Introduction

Small nucleolar RNAs (snoRNAs) are nucleolar non-coding RNAs which are a part of snoRNPs (ribosomal small nucleolar proteins) that function in 2’-O-ribose methylation and pseudouridylation cleavage reactions [1]. In plants, they are generally found to be organized as polycistronic clusters [2]. The snoRNAs complex, with a wide number of proteins, catalyze biogenesis and process ribosomal RNA in the nucleolus, as well as play a role as molecular chaperones [3,4]. Two known conserved classes of snoRNAs are box C (RUGAUGA)/D (CUGA) snoRNAs and box H (ANANNA)/ACA snoRNAs, the former C/D snoRNAs direct 2’-O-ribose methylation, while the latter H/ACA snoRNAs are involved in converting uridine to pseudouridine (ψ) [5]. Relatively more abundant snoRNAs (U3, U14, and 7-2/MRP) are highly conserved and transcribed from small nuclear RNA (snRNA) promoter elements in plants [6,7]. RNA polymerase III transcribes snoRNA [8], while U14 snoRNAs are clustered and transcribed as polycistronic transcripts [2]. It is known that snoRNAs also generate other small RNAs, such as sdRNAs (snoRNA-derived small RNAs) likely in response to stress [9,10]. In addition, in the case of tomato, early on, it was shown that snoRNAs also occur in tandem with genes coding for class I small heat shock proteins [11]. A role of snoRNAs in pre-rRNA processing in relation to ribosome biogenesis has been shown in Arabidopsis [12]. According to recent RNA deep sequencing and in situ localization studies, the function of snoRNA in ribosomal RNA (rRNA) modulation has been proposed [13].

Earlier, we discovered that class I small heat shock protein (hsp) genes in tomato were in tandem with the presence of two intronless snoRNAs, tomato box C/D *SlsnoRNA12.1* and *SlU24a* cluster, separated by a 105 nt spacer sequence mapped to tomato chromosome 6 [11]. Moreover, both tomato snoRNAs were found to have consensus C/D external (C and D) and internal (C’ and D’) sequence boxes along with two internal sequences complementary to ribosomal RNA [11]. Homologous D and E boxes, together with TATA-element sequence, were found present in the 5′ flanking region of snoRNA genes and, interestingly, a tandem repeat called homol-E was present in ribosomal protein gene promoters and known to act in proximal arrangement with homol-D as an activation sequence [11]. Moreover, we found that, during the ripening of tomato fruit, the levels of both *SnoR12.1* and *SnoU24a* maximized at the unripe, green stage of the fruit, declining thereafter upon ripening; during the progression of ripening, the decline in SnoR12.1 transcripts was slower than the rapid decline in SnoU24a transcripts [11]. Interestingly, an overall decline in protein synthesis accompanied by a decline in the de novo ribosome and rRNA synthesis was also previously observed in tomato fruit upon ripening [14,15,16].

The organization of tomato box C/D snoRNAs in concert with heat shock element (HSE) [11] has suggested the possibility of their functional role(s) in ribosomal RNA biosynthesis together with other proteins regulated by plant hormones and stress. In this context, the role of S-adenosylmethionine (SAM) in ribosome biogenesis and rRNA methylation has been shown in *Escherichia coli* [17]. Ribosomes, made of proteins and modified RNAs, carry out a critical function for cellular protein synthesis, which consumes enormous energy. The juxtaposition of snoRNA and sHsp clusters in concert with (HSE)-like elements in tomato [11] provides a system to unravel the regulation of rRNA, ribosome, and protein biogenesis in plants. In this context, another plant hormone group, a class of polyamines (spermidine and spermine), has emerged as hormones which, among other processes, also regulate RNA function(s) and protein synthesis [18]. Polyamines are ubiquitous plant growth regulators, among which only a few have been studied to some length; these include putrescine, spermidine, spermine, and thermo-spermine [19,20].

Tomato is a model for studying fruit ripening and unraveling interactions among plant hormones during ripening and senescence of tomato fruit [19]. Fruit ripening gaseous hormone ethylene has been considered to be an antagonist of polyamine action, and vice versa. To gain insight into the role of polyamines, i.e., spermidine (SPD) and spermine (SPM) in fruit ripening, a transgenic approach has been utilized to engineer a higher accumulation of SPD/SPM in tomato, only after the fruit have matured [21]. This strategy was successful and enhancement of the expression of a heterologous SAM decarboxylase gene led to utilization of PUT for the synthesis of SPD and SPM [21]. These studies also revealed that high polyamine fruit had a prolonged life span, higher keeping quality, and accumulated higher levels of the carotenoid lycopene [21]. Moreover, overaccumulation of higher polyamines in ripening transgenic tomato fruit also revived metabolic memory, upregulated anabolism-related genes, enhanced N:C signaling, and positively impacted nutritional quality [19].

We opined that, during ripening of an untransformed tomato, both snoRNA levels and those of SPD and SPM decrease and may be interlinked. It is known that polyamines, as well as nucleoli, are localized in the nucleus as also polyamine biosynthesis proteins, for instance, aminopropyltransferase [22]. Therefore, we utilized the transgenic tomato lines as a resource to address the question of interactive regulation between SPD/SPM and box C/D snoRNAs in upregulating ribosomal RNA biogenesis. Here, we demonstrate a clear nexus between polyamines SPD/SPM and clustered C/D box snoRNA gene transcription in concert with ribosomal RNA biogenesis in tomato.

## 2. Results

### 2.1. C/D Box snoRNAs in Tomato

**Figure 1:**
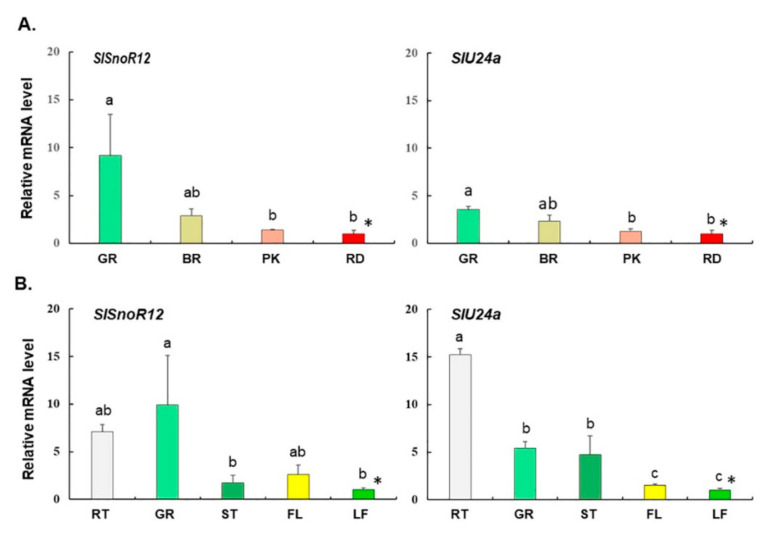
Quantitative RT-PCR of tomato fruit at different ripening stages (**A**) and of vegetative tissues (+ green fruit) (**B**). The values were normalized to the lowest value of either fruit ripening stage or the tissue type indicated by asterisks. (**A**) The fruit ripening stages, i.e., mature green (GR), breaker (BR), pink (PK), and red (RD). (**B**) Different tissue types, i.e., root (RT), mature green fruit (GR), stem (ST), flower (FL), and leaf (LF). The bars represent standard errors of mean (N = 3). The different letters above bars indicate statistical significance at *p* ≤ 0.05 as determined by Tukey’s test. * Represents the tissue used for normalization.

**Figure 2:**
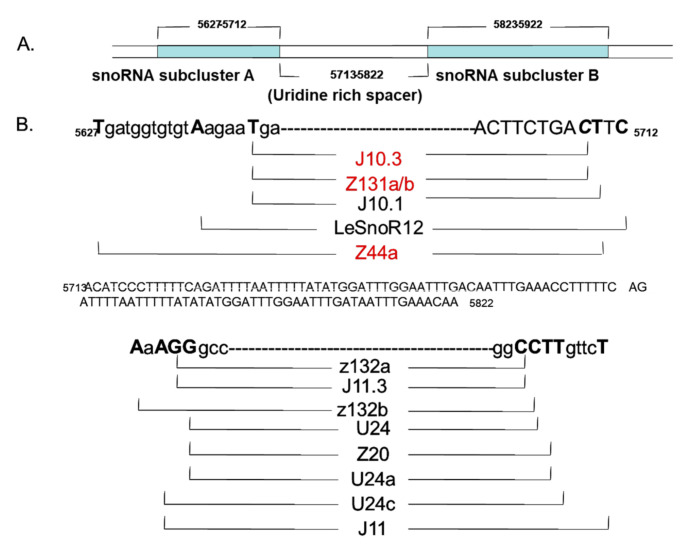
C/D box small nucleolar RNAs (snoRNAs) separated by a uridine rich spacer identified in tomato. Shown are snoRNA subcluster A (**A**) and snoRNA subcluster B (**B**) decorated with other C/D box snoRNAs, namely, J10.3, Z131a/b, J10.1, and Z44a followed by z132a, J11.3, z132b, U24, Z20, U24a, and J11. Several of these, for example, *SlZ44a*, *Slz132b,* and *SlU24a*, share conserved sequences with those in *Arabidopsis* and rice (Figure S2). For other details see [11].

We previously identified a cluster of class I small heat shock protein genes in tandem with a cluster of snoRNAs along with two box C/D RNAs, *SlsnoR12* and *SlsnoU24c* resident on chromosome 6 in tomato [11]. These snoRNAs were found to be also decorated with internal sequences complementary to ribosomal RNA [11]. The expression of both the *SlsnoR12* and *SlU24a* snoRNAs is highest in a mature green fruit (GR) and declines upon ripening of a normal, untransformed fruit (Figure 1A). Amongst the vegetative and other tissues, significantly higher expression of *SlU24a* was in the roots (RT) and much lower in stem (ST), leaf (LF), and flowers (FL) (Figure 1B). Similar to this snoRNA cluster housed on chromosome (Chr.) 6, two other noncoding C/D box genes, *SlsnoR12.2* and *SlU24b,* with a 94% identity to those on Chr. 6 were found located on Chr. 3 (Figure S1). Moreover, we also identified a number of other snoRNAs divisible into snoRNA subcluster A and snoRNA subcluster B, separated by a uridine rich spacer, and decorated with other C/D box snoRNAs, namely, J10.3, Z131a/b, J10.1, and Z44a followed by z132a, J11.3, z132b, U24, Z20, U24a and J11 (Figure 2). Several of these box C/D tomato snoRNAs, for example, *SlZ44a*, *Slz132b,* and *SlU24a*, share conserved sequences such as those in Arabidopsis and rice (Figure S2). *SlsnoU24* shares 79–82% identity with *Arabidopsis* U24a and rice *Z132a.b,* while *SlsnoR12* is 72–82% identical with *Arabidopsis snoR12* (*Z44a*) and rice (*Z131a.b* and *snoR12c*) (Figure S2).

### 2.2. Transgenic Tomato Fruit Lines with High Polyamine (SPD/SPM) Content Overexpress Box C/D snoRNA Transcription

**Figure 3:**
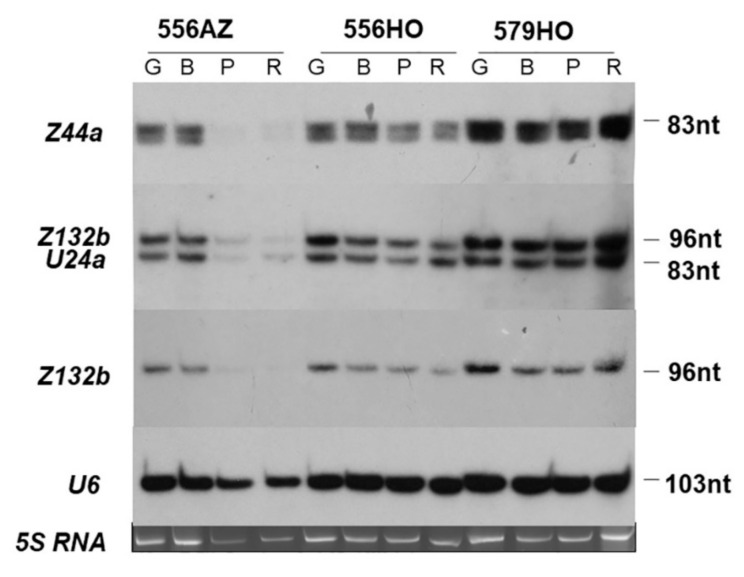
The snoRNA transcript profiles for azygous (556AZ) and high SPD/SPM transgenic (556HO and 579HO) tomatoes in green (G), breaker (B), pink (P), and red ripe (R–B +8) fruit. Profiles of the U6 control and 5S RNA are shown at the bottom.

Next, we analyzed the expression of a few box C/D snoRNAs, present in high SPD/SPM-accumulating tomato fruit (transgenic 556HO and 579HO lines), at different stages of ripening as compared with the non-transformed azygous 556AZ line (Figure 3). The Z44a, Z132b, and U24a snoRNAs in the non-transformed azygous 556AZ line were expressed in green and breaker stage fruit but their expression declined precipitously in pink and red stage fruit. In contrast, the expression of these same snoRNA genes in both the transgenic lines, 556HO and 579HO, remained consistently at a higher level at all stages from green to red ripe fruit. The U6 snoRNA, used as a control, remained more or less at the same level throughout the ripening of tomato. The fact that SIU24a maintained several-fold higher level in pink and red transgenic fruits as compared with the non-transgenic 556AZ line was further confirmed through qPCR (Figure S3). These data showed that polyamines SPD/SPM upregulated specific expression of these snoRNAs during ripening of the transgenic tomato fruit. For the next sets of experiments, we selected the transgenic line 579HO because its expression was more robust than the 556HO line and was in congruence with the profiles of the U6 control and 5S RNA (Figure 3).

### 2.3. Differential Expression of RNA Polymerase(s)/Transcription Genes in 556AZ and High SPD/SPM 579HO Tomato Lines

Our premise was that the endogenous overexpression of box C/D snoRNAs in high SPD/SPM tomato and longer life span of tomato fruit could partly be a reflection of upregulation of processes that finally led to ribosome biogenesis including ribosomal protein genes. RNA polymerases (Pol II, Pol III, and other), in tune with transcription factors, have been found to be critical for ribosome biogenesis [23] and references therein. Therefore, RNAseq of RNA from azygous (556AZ) and homozygous tomato line (570HO) at early breaker (B) and breaker+8 (B+8) stages were carried out to obtain a global picture of changes in RNA polymerases, ribosomal protein genes, translation initiation, and elongation factors. Annotation of 48 RNA polymerase-related genes revealed 17 DNA-directed RNA Pol II, III subunits along with other RNA Pol II, III transcriptional coactivators and mediators of transcription (Table S1).

The quantification of gene expression levels in 579HO as compared with 556AZ lines at the two fruit developmental stages (“B” and “B+8”) are presented as average FPKM (fragments per kilobase of transcript sequence per million base pairs) as well as ”differential gene expression log2 (fold change)”. The cutoff at 0.58 log2 fold change corresponds to 1.5 fold change in expression (Table 1, Table 2 and Table 3).

Thus, among the nine RNA polymerases shown in Table 1, expression of the majority of them increased from B to B+8 stage in both the 556AZ and 579HO lines, albeit the numbers were higher in the latter. The log2 (fold change) was significantly higher for seven genes at the B+8 stage, while two genes, namely, mediator of RNA Pol II transcription subunit 10 and RNA Pol II holoenzyme cyclin-like subunit were significantly downregulated. Notably, enrichment of RNA Pol II transcriptional coactivator p15, RNA Pol II, III subunits, DNA-directed RNA Pol II subunit, DNA-directed RNA Pol III, and RNA Pol II transcriptional coactivator p15 was apparent in the 579HO line at the B+8 ripe stage. At the early ripening B stage, the 579HO line was significantly upregulated only for RNA Pol II transcriptional coactivator p15 (Table 1).

### 2.4. Polyamines SPD/SPM as Anabolic Adjusters Upregulate Ribosomal Protein Genes

RNAseq analyses also revealed that, as compared with not so distinctive changes in RNA polymerase genes between 556AZ and 579HO lines (Table 1), among the 287 ribosomal proteins, 246 (86%) showed higher and 41 (14%) lower log2 fold change in the transgenic 579HO line at the B+8 stage as compared with the 556AZ control (Table S2). Among the 57 genes presented in Table 2, significant enrichment/upregulation of large subunit (L) members of ribosomal protein genes 60S (L5, L6, L7, L10, L13, L17, L22, L23, L24, L27, L31, L35, and L39), and 50S (L6, L7, L34, L33, and L35) as well as in the small subunit (S) members of ribosomal protein genes 40S (S6, S8, S10, S13, S18, S26, and S29) and 30S (S5 and S10) subunits in the 579HO line at the B+8 stage was distinctively apparent as compared with that in 556AZ at the B+8 stage (Table 2 and Table S2). The large 50S subunits (L6, L14, and L34) were also enriched at the early B stage of 579HO. Moreover, 50S ribosomal protein genes, namely, L1, L4, L7, L18, L27A, and L31, as well as of 40S (S6 and S26) were downregulated at both the ripening stages in the 579HO line. Many of these ribosomal protein genes (60S and 50S) in the 579HO line were complementary to one another, suggesting their coordinated regulation associated with polyamines SPD/SPM as compared with the control 556AZ line.

### 2.5. Polyamines SPD/SPM Upregulate Protein Translational Genes

Similar to the enrichment of ribosomal protein genes seen above, distinctly a good number of protein translation genes, namely, elongation factors, translation initiation factors, and translational activators were found to be upregulated at the B and B+8 stages in the transgenic 579HO line as compared with the 556AZ line (Table 3 and Table S3). Thus, among the 79 genes (Table S3), 31 were significantly and differentially expressed (Table 3). These included genes for elongation factors (for example, 1-alpha, 1-beta, 1-gamma, 1-beta guanine nucleotide exchange, elongation factor-like protein, and factor Tu), and translational initiation factors ( for example, factor 1 a, eIF1A, factor 2/2B, IF2/IF5, alpha subunit, gamma subunit; factor 3 subunits A, B, 2, 5, 6, 7, 8-like; factor 4, eIF4E; factor 5 IF2/IF5, 5A, subunit M, subunit 6, factor SUI1, pelota homolog; factor 5 IF2/IF5; eIF2B, IF3). Out of these, 28 were enriched at the B+8 stage in the transgenic 579HO line (Table 3). Moreover, and interestingly, a majority of the factors involved in general protein translation were either downregulated or did not show any differences at B and B+8 in the azygous 556 line (Table 3).

### 2.6. Pearson Correlations Among the RNA Polymerase(s), Ribosomal Protein(s), and Translation-Associated Protein Genes

**Figure 4:**
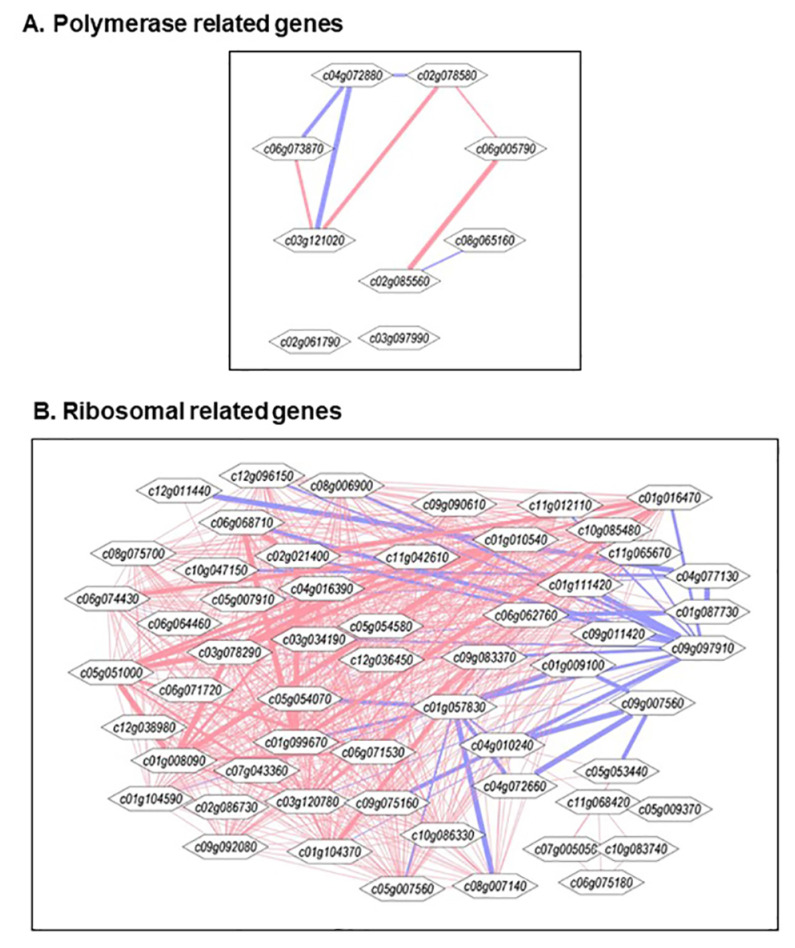

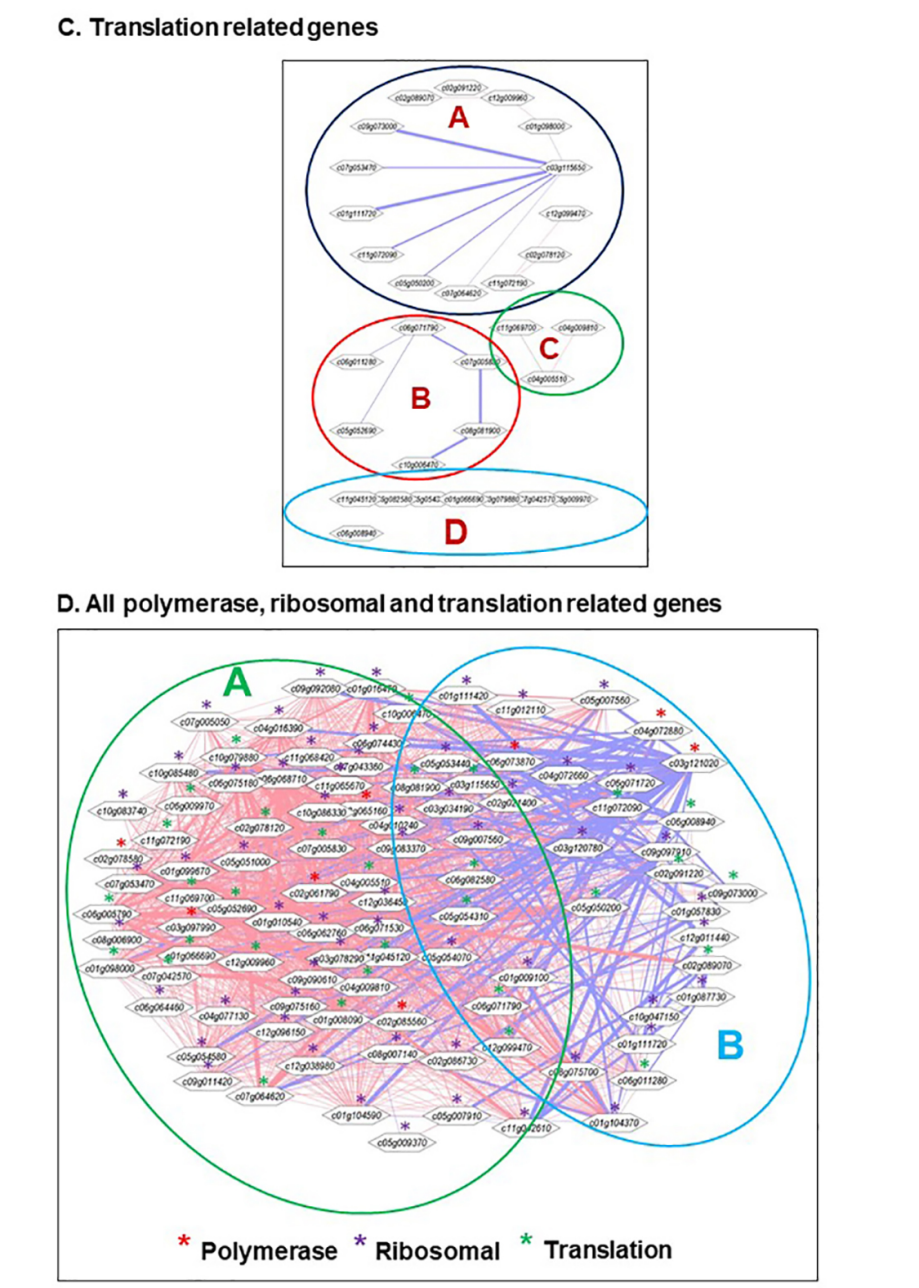
Cytoscape presentation of Pearson correlation coefficients among transcript levels of RNA polymerases (**A**), ribosomal biogenesis-related protein genes (**B**), translation machinery-related protein genes (**C**) and combined polymerase , ribosomal and translation genes (**D**).To determine the Pearson correlation among the polymerase, ribosomal and translation related genes, the FPKM of selected 9 polymerase (Table S4), 57 ribosomal (Table S6) and 31 translation (Table S5) were combined in a single Excel sheet and Pearson coefficient determined (Table S7). These correlations were visualized by Cytoscape (Figure 4D). Only correlation coefficients that exhibited values >0.9 and <−0.9 were used to determine the Metscape network. Networks were constructed using the Cytoscape-Metscape correlation-based build network program [24]. Red and blue edges indicate positive and negative correlations, respectively. The thickness of edges is proportional to relative correlation coefficient. The annotation of genes is the same as in Table S4. The values for all correlations are shown in Tables S5 and S6.

The Pearson correlations (±*r*) among polymerases, ribosomal protein, and translation-associated gene transcript levels were determined using EXCEL statistical package (Tables S4–S7). These ±*r* were profiled using Cytoscape to visualize expression networks for polymerases, ribosomal proteins, translation and combined genes, respectively, as shown in Figure 4A–D. Nine polymerase genes exhibited several ±*r* patterns (Figure 4A and Table S4). Five of these exhibited *+r* among them. RNA Pol II holoenzyme cyclin subunit (c04g072880) exhibited strong *−r* with c06g073870 (DNA-directed RNA Pol II subunit 4) and c03121020 (RNA Pol II-associated factor 1 homolog) (Table S4). Collectively, these results suggested that expression patterns of polymerase genes differed significantly from one another with some exhibiting no correlation among them. We interpret these results to suggest that snoRNAs do not have uniformly coordinate expression of polymerase genes.

The 31 translation-related genes profiled contained 21 TIFs (TIF) and nine elongation factors (EF) (Figure 4c and Table S5). According to Pearson ±*r,* they were broadly divided into four clades. Clade A contained the maximum number of genes, several of which exhibited very strong *−r*. Solyc03g115650 (TIF 5A) showed strong *−r* with some of them, especially strong with EF-Tu (Solyc09g073000) and TIF-3 (Solyc01g111720). TIF 5A also exhibited strong *−r* with EF-A (Solyc07g053470), EF beta-1 (Solyc11g072190) and TIF-1A (Solyc05g050200). Several weak positive correlations were also observed in Clade A (Figure 4C). Clade B contained six translation-related genes, and all exhibited *−r* with at least one another gene. EF-Tu (Solyc06g071790), TIF-3 (Solyc07g005830), TIF-2 (Solyc08g081900) and TIF-SUI1 (Solyc10g006470) showed particularly strong *−r* among them. Clade C, which had three translation genes, exhibited weak ±*r* among them. Eight genes in Clade D did not exhibit ±*r* in range exhibited values > 0.9 and <−0.9 (Figure 4C and Table S5). Taken together, these results suggest that most translation genes exhibit independent patterns.

The Cytoscape network for gene expression of 57 ribosomal genes afforded a large number of genes (Table S6 and Figure 4B). This set contained two 30S, six 40S, thirteen 50S, twenty-five 60S, and fourteen other ribosomal protein genes, respectively (Table S6). Pearson correlations (±*r*) between these ribosome-related genes were determined and profiled for the Cytoscape network (Figure 4B). These genes could be broadly divided into the following three categories: (1) strong +*r*, (2) strong −*r,* and (3) weak to little *r* (Figure 4C and Table S6). Ribosomal genes that exhibited strong +r included 30S RP S19, 40S RP S10-like, 40S RP S13, 40S RP S13, 40S RP S28, 40S RP S5, 40S RP S7-like, 50S RP L14, 50S RP L34, 50S RP L7Ae, 50S RP L7Ae, 4S RP L39, 60S acidic RP L10, 60S acidic RP P1, 60S acidic RP-like, 60S RP, 60S RP L13, 60S RP L13, 60S RP L18a, 60S RP L22-2, 60S RP L23a, 60S RP L24, 60S RP L27, 60S RP L32, 60S RP L34, 60S RP L37, 60S RP L37a, 60S RP L44, 60S RP L6, 60S RP L6, RP L1, RP L12, RP L3, RP PSRP-3, RP S27, RP S27, and RP S9 (Figure 4C and Table S4). Ribosomal genes that exhibited strong −*r* included 30S RP S1, 50S RP L18, 50S RP L5, RP S1, and RP L. The ribosomal genes that exhibited weak to no ±*r* included 60S RP L30, 50S RP L1b, 50S RP L12-C, 60S RP L35, 60S RP L30e, 50S RP 6, 50S RP L15b, 60S RP L29, RP L7a, 60S RP L27a, RP L12, 60S RP L39e, 50S RP L4, and 60S RP L24e (Figure 4B and Table S6). We interpret these results to suggest that ribosomal genes exhibit variable and strong regulation of coordinate expression.

Coordinate regulation of expression among the polymerase, translation, and ribosomal genes analyzed are shown in Figure 4D. The Cytoscape network distribution of these genes can be broadly divided into two clades. Clade A represented most genes that exhibited generally +*r* with other genes and Clade B contained genes that showed generally −*r* with genes in this clade. All the three types of genes, i.e., polymerase, ribosomal, and translation, were scattered throughout the Cytoscape network, suggesting the presence of a mixed intra- and intercoordinate regulation of these genes. The ribosomal genes exhibited coordinate expression with polymerase genes and to a limited extent with translation genes (Figure 4D and Table S7).

### 2.7. Specific Enrichment of Transcriptome as a Result of Transgene and Developmental Stage

**Figure 5:**
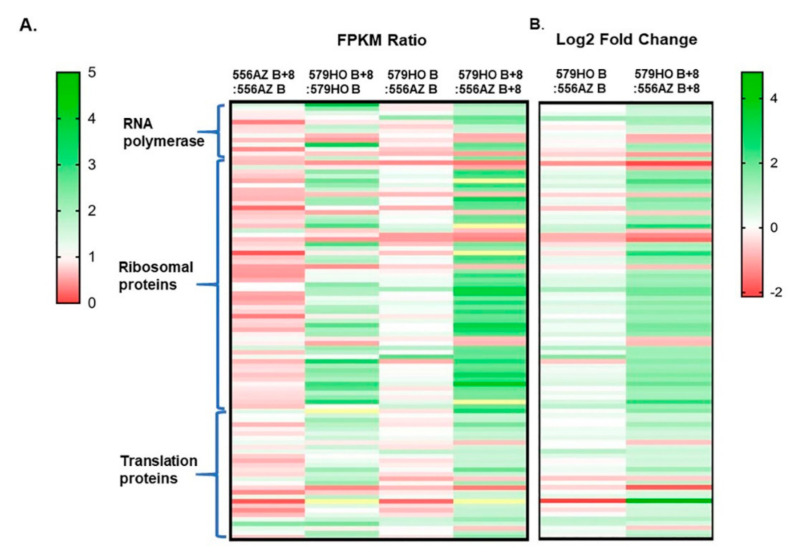
Heatmaps of gene expression analysis of the identified RNA polymerases, ribosomal proteins, and translation protein genes in 556AZ and 579HO lines at breaker (B) and breaker+8 (B+8) stages. Panel (**A**) shows FPKM expression ratios at B and B+8 stages for 556AZ B+8:556AZ B and 579HO B+8:579HO B and between two lines (579HO B:556AZ B and 579HO B+8:556AZ B+8). Panel (**B**) highlights “differential” expression as log2 fold change in 579HO line as compared with 556AZ at two ripening stages. Heatmaps were performed using GraphPad Prism version 7.0 for Windows, GraphPad Software, La Jolla, CA, USA, www.graphpad.com.

The relative expression of a transcript is proportional to the number of cDNA fragments that originate from it. Therefore, we used normalized FPKM (Figure 5A) to visualize differential and gene-specific related expression of protein biogenesis genes (RNA polymerase, ribosomal, and translation protein genes) and these are presented in Table 1, Table 2 and Table 3. The FPKM expression comparisons of 97 genes at B and B+8 stages for 556AZ B+8/556AZ B and 579HO B+8/579HO B revealed that polyamines SPD/SPM upregulate specific gene members at the B+8 ripe stage versus the B stage as compared with the patterns seen in the azygous 556 line (Figure 5A). The dominant effect of the developmental stage in the genotype 579HO by comparing 579HO B/556AZ B and 579HO B+8/556AZ B+8 showed increased average FPKM values as well as log2 fold change. The ratio between the two stages and between the two lines highlights the fact that SPD/SPM regulate a specific and unique set of tomato protein synthesis-related genes at the red ripe stage (B+8) not observed with the 556AZ control line (Figure 5A,B).

### 2.8. Proteins Associated with the Ribosomal Exit Tunnel

**Figure 6:**
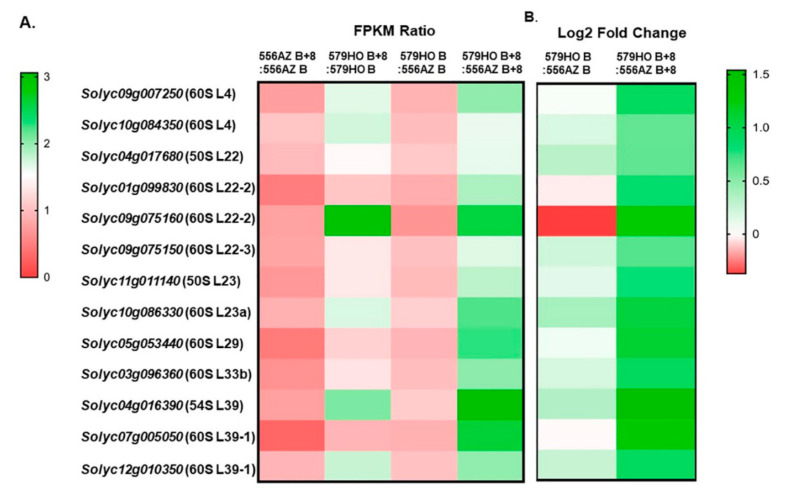
Heatmaps of gene expression analysis of the identified ribosome exit tunnel protein genes in the 556AZ and 579HO lines at breaker (B) and breaker+8 (B+8) stages. (**A**) FPKM expression ratios of 556AZ B+8:556AZ B and 579HO B+8:579HO B at B vs. B+8 stages, and between 579HO B:556AZ B and 579HO B+8:556AZ B+8). (**B**) Differential expression (log2 fold change) in 579HO as compared with 556AZ at B vs. B+8 ripening stages. Heatmaps were performed using GraphPad Prism version 7.0 for Windows, GraphPad Software, La Jolla, CA, USA (www.graphpad.com).

The ribosome exit tunnel is considered to be a platform that regulates translation and folding of proteins, chaperones, and several other factors to enable proper emergence of nascent polypeptides via the exit tunnel [25,26]. A number of ribosomal proteins are known which specifically coordinate along the nascent chain in the tunnel at different points. Some of these are named L4, L22, L23, and L29. We identified, in tomato, similar and other sets of proteins that are known to coordinate along the ribosomal exit tunnel. Expression values (FPKM ratio) and differential levels of expression (log2 fold change) for the identified ribosome exit tunnel protein genes, namely, 60S: L4, L22-2, L22-3, L23a, L29, L33b, and L39-1; and 50S: L22, L23, and L39 are presented in Figure 6A,B. The expression of ribosomal exit tunnel genes in the 556AZ line was downregulated from the B to the B+8 stage (Figure 6A) but distinctly upregulated in the 579HO line at the B+8 stage (Figure 6A,B).

## 3. Discussion

One major biogenetic process involved in the regulation of transcription and processing of pre-mRNA complexes in the nucleus involves the role of small nucleolar RNAs (snoRNAs). A role of snoRNAs in pre-rRNA processing in relation to ribosome biogenesis has become apparent from RNA deep sequencing and in situ localization studies in Arabidopsis [12,13]. The organization of tomato box C/D snoRNAs in concert with heat shock elements suggested the possibility of their functional role(s) in ribosomal RNA biosynthesis together with other proteins regulated by plant hormones and stress [11], apparent also by the role(s) of S-adenosylmethionine (SAM) in ribosome biogenesis and rRNA methylation shown in *Escherichia coli* [17]. SAM is an essential substrate for the biosynthesis of polyamines (spermidine and spermine) that have emerged as plant hormones, which were previously thought to regulate RNA function(s) as well as protein synthesis [18]. In contrast to what was observed in the azygous tomato, the steady-state levels of several snoRNAs did not decline in the high SPD/SPM transgenic fruits upon progression of ripening (Figure 3). Instead, the snoRNA levels remained relatively at either the same level or higher as at the early stages of fruit development in the transgenic 579HO ripe fruits (Figure 3). Since the 556AZ line and transgenic 579HO are isogenic, these results provide genetic evidence for the role of SPD/SPM in the synthesis and accumulation of snoRNAs in tomato fruits. These results are consistent with previous studies where SPD/SPM were implicated in restoring and enhancing metabolic activities at the levels of mRNA, proteins, and enzymatic activities in tomato [27,28,29].

The dramatic decline in the snoRNA levels of the azygous tomato, shown here, indicates the loss of biosynthetic machinery leading to an irreversible phase of fruit development that encompasses senescence followed by disintegration and decay of the fruit. The association between the decline in snoRNAs and fruit senescence suggests that these two events are likely connected, with the snoRNA decline likely acting as a signal for seed dispersal. This interpretation is in tune with the growing body of literature showing that snoRNAs regulate a multitude of biological processes such as post-transcriptional modification of RNA, including acetylation, regulation of splicing patterns, stability and control of mRNA abundance, and translation [13,30,31].

The data presented here, including the RNAseq analyses, clearly demonstrate the significant enrichment of specific RNA polymerase, ribosomal, and translational protein genes in high SPD/SPM transgenic tomato at the red ripe (breaker+8) stage as compared with the 556AZ control. Thus, these results indicate that SPD/SPM regulate snoRNA and rRNA expression and, in turn, affect protein synthesis, metabolism, and other cellular activities in a positive manner. A good number of snoRNAs are now known in several plant species, including rice, Arabidopsis, and tomato [11,32,33,34]. However, in spite of the abundant evidence for role(s) of snoRNAs in plants, the molecular basis of snoRNA effects have not yet been fully unraveled. A role for snoRNA in ribosome biogenesis has been suggested, but detailed characterization has not been determined [4,35]. Transcription, translation, and protein synthesis are complex and highly regulated processes in eukaryotes, including plants. Here, we show that snoRNAs are associated with upregulation of genes for a large number of RNA polymerases, ribosomal proteins, translational machinery proteins, and those involved in the ribosome exit tunnel (Figure 4, Figure 5, Figure 6 and Table 1, Table 2, Table 3). Most of these proteins were found coordinately regulated and exhibited correlation coefficient > 0.9 (Figure 4 and Tables S5–S7). However, a small number of genes did not show high (>0.9) correlation co-efficiency and separated out as a different subgroup (Figure 4). Thus, our interpretation of these data is that snoRNAs have enormous involvement in regulating growth and development in living organisms including plants by effecting transcription of “specific” groups of genes and gene products.

The Pearson correlation coefficient among the polymerase, ribosomal, and translation-related gene expression provides novel information. Most significant among which is that steady-state regulation of genes from the three classes, i.e., polymerase, ribosomal, and translation, is not similar in response to changes in snoRNA and Spd/Spm levels in the ripening tomato fruits (Figure 4 and Tables S4–S7). A large number of ribosomal genes (37 out of 56 ribosomal genes exhibited strong *r* (>0.9) with each other indicating coordinate regulation of expression. The 36 ribosomal genes that exhibited coordinate regulation also showed strong *r* (>0.9) with 18 out of 31 translation related genes. The remaining 19 ribosomal genes and 13 translation genes showed lower inter and intra association with *r* (<0.9 or >−0.9). These data indicate that snoRNA- and Spd/Spm-regulated gene expression represent two tier control. Expression of a set of these genes is coordinately regulated in the high snoRNA/Spd/Spm fruits, whereas other sets within the three categories are independent of the snoRNA/polyamine levels. This differential expression pattern would likely have ramifications in how these plants modulate gene expression under normal conditions versus under, for example, stress perceived by the plant. The nexus of high SPD/SPM, together with higher expression of the snoRNAs, opens a path for new direction to understand the regulation of polymerase, ribosomal, and translation machinery in playing significant roles in different aspects of fruit ripening, prolonged life span, and perhaps in plants’ adaptability to different abiotic stresses. Thus, our interpretation of these data is that snoRNAs have enormous involvement in regulating growth and development in living organisms including plants by effecting transcription of “specific” groups of genes and gene products.

**Figure 7:**
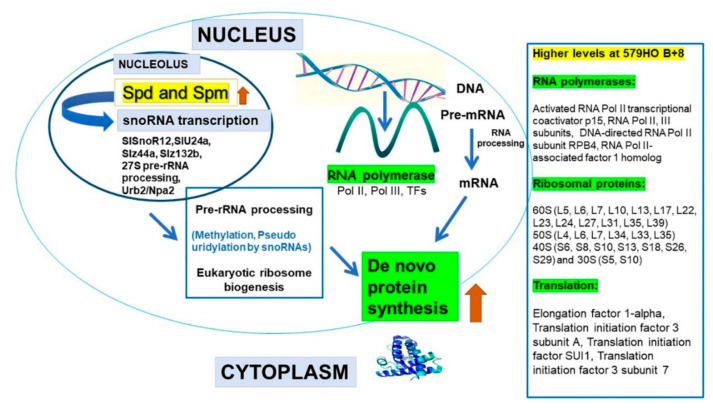
SPD/SPM upregulate nucleolar snoRNAs, initiating RNA processing and maturation leading to massive protein synthesis.

Studies on chloroplast transition into chromoplast in tomato fruit have previously identified metabolic shifts in the proteome, as well as a decline in the overall rate of RNA synthesis [36,37]. In these studies, functional ribosomes and translational activity were related to the chromoplast function at later stages of ripeness in tomato where minimal polyamine levels were seen. As seen in the present study, the SPD/SPM tomato fruit have a higher overall synthesis of RNA and are also predominant in genes related to ribosomal proteins, translation proteins, and ribosomal exit tunnel-specific proteins (Figure 5, Figure 6 and Figure 7). Thus, we suggest that chromoplast function may be active at a higher dimension in high SPD/SPM tomato due to specific and high upregulation of the proteome-related genes.

The demonstration, here, of the specific alteration in the protein translational machinery in the transgenic tomato fruit that specifically express and accumulate growth regulators SPD and SPM also provided new information about the regulation of specific elongation factor-1 alpha (eIF1) genes (Table 3). EF1 alpha mRNA is abundantly present in developing young leaves and green tomato fruit [38] but declines precipitously in red fruit [38,39]. On the one hand, a diminished level of EF1 alpha has been considered to be a part of physiological processes associated with fruit development and programmed cell death. Although not fully characterized in plants, eIF3A has been suggested to be strictly regulated [39]. On the other hand, a senescing tomato fruit undergoing programmed cell death has been shown to be associated with an increase in eIF5A level [40]. In the context of our findings here, it does not appear to be a mere coincidence that EF1 alpha, eIF3 and eIF3A genes were maintained at significantly higher levels, while eIF5A was downregulated in SPD/SPM-enriched transgenic red tomato fruit (Table 3). Thus, our findings, though correlative, strongly suggest the involvement of SPD/SPM in altering the protein translation machinery to keep the fruit developmental process active for a longer time period [21,27].

Research on snoRNAs in tomato is scarce. A notable study on tomato pollen detected seven snoRNAs in MACE (massive analyses of cDNA ends library) and their response to heat stress [41]. These authors concluded that since plant pre-snoRNA were not spliced, therefore, snoRNAs could regulate protein synthesis and plant response to stress. Here, based on our results, we propose a model (Figure 7) in which nexus between snoRNAs and SPD/SPM in the nucleolus initiates pre-rRNA processing and steady-state polymerase genes that possibly generate endogenous regulatory signals leading to initiation of ribosomal protein(s) biogenesis coordinated with transcription, elongation/initiation factors. In turn, the maturation/ upregulation of ribosome exit tunnel genes leads to massive protein synthesis and stimulation of biogenic processes to enable, in this instance, a ripening fruit to have a longer shelf life and maintain higher levels of carotenoids (lycopene) and secondary metabolites, as previously established with the high SPD/SPM tomatoes [19,21,27,28,29].

## 4. Materials and Methods

### 4.1. Plant Material, Sample Preparation, and Replications

Tomato (*Solanum lycopersicum* L. cv. Ohio 8245) azygous control (556AZ) and homozygous transgenic tomato line (579HO; 556HO) were grown in a temperature-controlled greenhouse at Beltsville Agricultural Research Center (Maryland, USA) under natural light conditions [21]. For initial determination of C/D box snoRNA levels, tomato fruit at different stages of ripening (green (GR), breaker (BR), pink (PK), and red ripe (RD)) and vegetative/fruit tomato tissues (root (RT), green fruit (GR), stem (ST), leaf (LF) and flower (FL)) were analyzed, as previously described [11] (Figure 1, Figure 2 and Figure 3). For RNAseq analysis, three biological replicates (3 fruits per replicate) sampled at breaker (B) and breaker+8 (B+8) stages were used. Pericarp tissue was sampled and frozen immediately in liquid nitrogen and kept at −80 °C until used for analysis, as well as for RNA isolation.

### 4.2. Cloning and DNA Sequencing

Isolation of total RNA and cloning of tomato snoRNAs have been described in detail previously [11]. 

### 4.3. Total RNA Extraction, cDNA Template Preparation, and Q-PCR Analysis

Total RNA was extracted from three biological replicates of tomato pericarp fruit tissue (at defined stages of ripening), secondary roots, secondary stems, young leaves, and flowers and quantified by nanodrop, as previously described [11]. Samples with a A260/280 ratio of 1.9:2 were subjected to agarose gel electrophoresis to ensure the presence of intact rRNA bands. Methods used for total RNA reverse transcription, Q-PCR, and data analysis (delta-delta CT method) were essentially the same as described before [12]. The primers used for Q-PCR and Northern are listed in Table S8. The PCR conditions were 95 °C for 30 s, 55 °C for 30 s, and 72 °C for 30 s, followed by melt curve analysis.

### 4.4. RNA Isolation and Sequencing

Total RNA was isolated from powdered tomato fruits of three biological replicates, each containing pooled pericarps from three fruits using a Plant RNeasy kit (QIAGEN). RNase-Free DNase (QIAGEN) was used to remove genomic DNA followed by a cleanup with a RNeasy Mini Kit (QIAGEN). RNA degradation and contamination were monitored using 1% agarose gels. The RNA purity was checked using the NanoPhotometer^®^ spectrophotometer (IMPLEN, Westlake Village, CA, USA). RNA integrity and quantification were assessed using the RNA Nano 6000 Assay Kit of the Bioanalyzer 2100 system (Agilent Technologies, Carpinteria, CA, USA). The RNA samples, with RIN values between 7 and 9, were used for the RNA sequencing using 125/150 bp paired end read mode with Illumina HiSeq2000 platform by Novogene Bioinformatics Technology Co., Ltd., in Sacramento, CA, USA. A total amount of 1 μg RNA per sample was used as input material for the RNA sample preparations. Sequencing libraries were generated using NEBNext^®^ Ultra™ RNA Library Prep Kit for Illumina^®^ (NEB, Ipswich, MA, USA). Briefly, mRNA was purified from total RNA using poly-T oligo-attached magnetic beads. After mRNA fragmentation and cDNA synthesis, cDNA fragments of preferentially 150~200 bp in length, the library fragments were purified with the AMPure XP system (Beckman Coulter, Beverly, MA, USA) and library quality was assessed using the Agilent Bioanalyzer 2100 system.

### 4.5. Transcriptome Analysis

The clustering of the index-coded samples was performed on a cBot Cluster Generation System using PE Cluster Kit cBot-HS (Illumina), according to the manufacturer’s instructions. The raw reads of fastq format were firstly processed through in-house perl scripts. In this step, clean reads were obtained by removing reads containing adapter, reads containing ploy-N, and low-quality reads from raw data. In addition, Q20, Q30, and GC content clean data were calculated. All the downstream analyses were based on the clean data with high quality. For mapping reads, the reference genome was built using HISAT2 (2.1.0) and paired-end clean reads were aligned to the reference tomato (*S. lycopersicum*) genome and ITAG4.0 version of the annotation (tomato genome/annotation/ITAG4.0). Fragments per kilobase million (FPKM) mapped reads were used to estimate the gene expression levels [36]. For quantification of gene expression levels, HTSeq v0.6.1 was used to count the number of reads mapped to each gene. The FPKM (fragments per kilobase of transcript sequence per million) of each gene was calculated based on the length of the gene and read counts mapped to this gene. FPKM, i.e., the expected number of base pairs sequenced, considers the effect of sequencing depth and gene length for the read counts at the same time, and is currently the most commonly used method for estimating gene expression levels [42].

Differential expression analysis of 579HO versus 556AZ at two fruit developmental stages (three biological replicates per stage) was performed using the DESeq R package (1.18.0) [43]. DESeq provided statistical routines for determining differential expression in digital gene expression data using a model based on the negative binomial distribution. The resulting p-values were adjusted using the Benjamini and Hochberg’s approach [44] for controlling the false discovery rate (FDR). Genes with an adjusted *p*-value < 0.05 found by DESeq were considered to be significant differentially expressed. The genes related to RNA polymerase, ribosomal, and translation proteins were analyzed.

Differences among treatments were considered to be significant at *p* < 0.05. The heatmap, correlation and Cytoscape analyses were performed using GraphPad Prism (7.0), Excel, and Cytoscape [45] programs, respectively. The networks were built based on correlations using Cytoscape version 3.8.1.

## Figures and Tables

**Table 1:** Differential expression of RNA polymerase genes in 556AZ and 579HO tomato fruit at breaker (B) and breaker+8 (B+8) stages.

		Avg FPKM in 556AZ and 579HO Fruits *				579HO B vs. 556AZ B		579HO B8 vs. 556AZ B8	
Gene ID	Gene Description	556AZ B	556AZ B8	579HO B	579HO B8	log2 Fold Change	padj	log2 Fold Change	padj
Solyc02g085560	DNA-directed RNA Pol III	2.85	4.60	2.48	10.24	−0.02	1.00	**1.07**	***4.00E−03***
Solyc06g005790	DNA-directed RNA Pol II subunit	16.80	19.62	14.89	33.99	0.01	1.00	**0.71**	***6.90E−05***
Solyc06g073870	DNA-directed RNA Pol II subunit 4	78.52	70.75	84.79	118.61	0.30	0.69	**0.67**	***7.10E−06***
Solyc02g061790	Activated RNA Pol II transcriptional coactivator p15	24.71	22.06	56.88	58.29	1.38	***1.90E−05***	**1.32**	***4.60E−13***
Solyc03g097990	RNA Pol II subunit	18.16	5.70	16.28	14.19	0.04	1.00	**1.23**	***3.40E−05***
Solyc02g078580	RNA Pol-associated RTF1 homolog	15.85	12.62	11.96	21.23	−0.21	0.87	**0.67**	***9.10E−05***
Solyc03g121020	RNA Pol II-associated factor 1 homolog	10.84	8.99	9.88	14.69	0.05	1.00	**0.63**	***5.50E−04***
Solyc04g072880	RNA Pol II holoenzyme cyclin subunit	13.95	16.45	14.35	9.06	0.21	0.88	**−0.94**	***4.10E−06***
Solyc08g065160	Mediator of RNA Pol II transcription subunit 10	95.00	60.94	78.75	36.14	−0.09	1.00	**−0.83**	***1.60E−07***

The cutoff is 0.58 log2 fold change at 579HO B8 vs. 556AZ B8. The significant log2 fold changes are in bold font. The significant padj ≤ 0.05 are in bold and italics. * Average of three independent biological replicates. Green color cells show positive values and orange color cells show negative log2 fold change in 579HO as compared with 556AZ.

**Table 2:** Differential expression of ribosomal protein in 556AZ and 579HO tomato fruit at breaker (B) and breaker+8 (B+8) stages.

		Avg FPKM in 556AZ and 579HO Fruits *				579HO B vs. 556AZ B		579HO B8 vs. 556AZ B8	
Gene ID	Gene Description	556AZ B	556AZ B8	579HO B	579HO B8	log2 Fold Change	padj	log2 Fold Change	padj
Solyc01g111420	RP L1	19.11	23.38	17.86	65.16	0.10	1.00	**1.40**	***4.60E−22***
Solyc11g068420	RP L1	289.50	107.82	207.49	263.92	−0.28	0.81	**1.21**	***7.00E−19***
Solyc01g087730	50S RP L1b	90.04	81.22	55.64	38.62	−0.51	0.24	−1.15	***4.90E−16***
Solyc01g104590	RP L3	296.25	204.70	303.40	528.49	0.23	0.80	**1.29**	***3.40E−24***
Solyc10g047150	50S RP L4	9.10	5.74	3.34	1.43	−1.28	0.37	−2.08	***0.019***
Solyc09g007560	50S RP L5	55.13	85.42	53.18	46.52	0.13	0.97	−0.96	***2.60E−11***
Solyc11g012110	60S RP L6	300.29	238.37	315.34	732.88	0.27	0.74	**1.54**	***4.80E−33***
Solyc05g054070	60S RP L6	94.40	66.36	119.59	191.29	0.54	0.23	**1.45**	***5.90E−27***
Solyc09g092080	50S RP L7Ae	11.94	6.39	12.62	33.37	0.28	0.95	**2.3**	***2.00E−20***
Solyc09g011420	50S RP L7Ae	33.05	32.45	44.31	98.11	0.64	0.21	**1.52**	***4.90E−22***
Solyc12g038980	50S RP L7Ae	153.52	98.20	143.56	240.65	0.12	1.00	**1.21**	***1.90E−17***
Solyc06g064460	RP L7a	52.69	33.24	32.60	21.25	−0.5	0.28	−0.73	***3.40E−05***
Solyc05g054580	60S acidic RP L10	205.85	121.41	201.33	431.54	0.17	0.92	**1.75**	***3.30E−15***
Solyc11g065670	RP L12	238.64	200.29	257.42	548.15	0.31	0.66	**1.37**	***4.60E−25***
Solyc06g075180	RP L12	420.22	88.61	237.59	238.75	−0.63	0.18	**1.35**	***4.00E−21***
Solyc02g086730	50S RP L12-C	86.37	59.54	82.09	41.44	0.13	0.99	−0.6	***9.70E−04***
Solyc08g075700	60S RP L13	246.14	184.16	276.56	489.11	0.37	0.53	**1.33**	***1.10E−24***
Solyc12g096150	60S RP L13	131.14	108.59	134.72	271.45	0.24	0.82	**1.24**	***7.30E−20***
Solyc09g090610	50S RP L14	76.33	51.01	106.92	310.40	0.71	0.37	**2.53**	***3.50E−26***
Solyc05g009370	50S RP L15b	20.68	35.38	32.93	23.83	**0.85**	***0.02***	−0.65	***1.70E−04***
Solyc12g011440	60S RP L18a	35.57	35.24	16.54	14.60	**−0.93**	***0.03***	−1.35	***3.40E−10***
Solyc05g007910	50S RP L18	3.14	2.06	1.49	0.67	−0.87	0.45	−1.71	***0.006***
Solyc09g075160	60S RP L22-2	23.36	17.96	15.57	46.49	−0.37	0.71	**1.29**	***1.30E−09***
Solyc10g086330	60S RP L23a	107.77	94.18	122.15	208.20	0.39	0.76	**1.06**	***3.30E−13***
Solyc10g083740	60S RP L24e	57.96	5.96	39.27	35.59	−0.35	0.66	**2.50**	***1.40E−25***
Solyc10g085480	60S RP L24	373.57	257.75	422.28	764.50	0.37	0.50	**1.49**	***1.90E−30***
Solyc07g043360	60S RP L27	359.28	277.37	383.98	754.59	0.30	0.66	**1.36**	***4.40E−25***
Solyc06g071720	60S RP L27a	2125.46	1035.99	1619.17	648.79	−0.21	0.82	−0.76	***6.30E−09***
Solyc05g053440	60S RP L29	176.57	82.63	161.86	187.73	0.07	1.00	**1.11**	***4.80E−14***
Solyc04g072660	60S RP L30e	139.80	61.67	155.02	183.65	0.35	0.59	**1.49**	***3.40E−26***
Solyc01g009100	60S RP L30	58.76	34.65	71.41	86.61	0.49	0.58	**1.24**	***1.80E−16***
Solyc08g006900	60S RP L32	20.78	20.66	24.46	56.71	0.44	0.72	**1.38**	***5.90E−20***
Solyc06g062760	50S RP L34	4.93	5.20	10.20	19.76	**1.23**	***0.01***	**1.85**	***4.20E−16***
Solyc05g007560	60S RP L34	151.76	81.29	162.80	307.86	0.30	0.77	**1.84**	***5.70E−42***
Solyc04g010240	60S RP L35	165.20	78.68	159.67	192.83	0.15	0.96	**1.21**	***5.20E−18***
Solyc08g007140	60S RP L37a	208.80	118.69	270.08	368.63	0.58	0.58	**1.56**	***5.20E−30***
Solyc03g120780	60S RP L37	173.59	150.63	195.36	378.27	0.37	0.54	**1.25**	***9.90E−21***
Solyc04g016390	54S RP L39	19.73	14.91	21.93	45.63	0.35	0.88	**1.54**	***3.30E−07***
Solyc07g005050	60S RP L39e	342.13	104.92	299.71	275.44	−0.01	1.00	**1.31**	***1.00E−21***
Solyc06g071530	60S RP L44	137.29	109.19	171.14	296.82	0.52	0.23	**1.36**	***2.60E−23***
Solyc01g016470	60S RP	41.35	31.00	40.77	114.70	0.18	0.94	**1.81**	***1.80E−32***
Solyc06g074430	60S acidic RP-like	316.74	183.27	316.94	623.50	0.20	0.87	**1.69**	***2.30E−38***
Solyc01g104370	60S acidic RP P1	269.11	248.17	337.20	712.90	0.53	0.22	**1.44**	***6.00E−29***
Solyc01g057830	30S RP S1	24.71	28.76	21.80	19.04	0.01	1.00	−0.67	***7.80E−05***
Solyc09g097910	RP S1	9.30	8.50	9.76	5.16	0.25	0.83	−0.8	***0.001***
Solyc03g034190	RP PSRP-3	14.10	21.85	26.37	57.73	**1.09**	***3.30E−03***	**1.32**	***7.70E−11***
Solyc11g042610	40S RP S5	76.76	52.94	77.27	142.69	0.22	0.92	**1.35**	***4.00E−20***
Solyc04g077130	50S RP 6	47.48	58.64	136.63	148.10	**1.72**	***1.50E−08***	**1.26**	***2.80E−12***
Solyc12g036450	40S RP S7-like	2.72	1.64	1.55	5.18	−0.66	0.93	**1.58**	***2.60E−02***
Solyc01g010540	RP S9	62.19	49.36	65.77	128.93	0.29	0.77	**1.31**	***4.90E−20***
Solyc01g099670	40S RP S10-like	182.66	152.99	178.48	430.71	0.17	0.93	**1.41**	***1.30E−27***
Solyc03g078290	40S RP S13	86.40	59.22	94.15	205.97	0.32	0.65	**1.72**	***4.70E−07***
Solyc05g051000	40S RP S13	35.61	27.55	40.87	78.99	0.41	0.71	**1.44**	***6.60E−20***
Solyc09g083370	30S RP S19	1.97	1.88	2.66	7.98	0.63	0.68	**2.01**	***1.10E−08***
Solyc06g068710	RP S27	9.11	7.89	7.72	21.17	−0.03	1.00	**1.34**	***1.50E−09***
Solyc01g008090	RP S27	36.38	29.68	40.39	73.07	0.36	0.67	**1.22**	***4.70E−13***
Solyc02g021400	40S RP S28	549.84	437.62	494.04	1117.90	0.05	1.00	**1.27**	***4.60E−13***

The cutoff is 0.58 log2 fold change at 579HO B8 vs. 556AZ B8. The significant log2 fold changes are in bold font. The significant padj ≤ 0.05 are in bold and italics. * Average of three independent biological replicates. Green color cells show positive values and orange color cells show negative log2 fold change in 579HO as compared with 556AZ.

**Table 3:** Differential expression of translation protein genes in 556AZ and 579HO tomato fruit at breaker (B) and breaker+8 (B+8) stages.

		Avg FPKM in 556AZ and 579HO Fruits *				579HO B vs. 556AZ B		579HO B8 vs. 556AZ B8	
Gene ID	Gene Description	556AZ B	556AZ B8	579HO B	579HO B8	log2 Fold Change	padj	log2 Fold Change	padj
Solyc07g064620	TIF SUI1	228.98	168.15	301.90	967.76	0.58	0.13	**2.45**	***1.22E−05***
Solyc10g006470	TIF SUI1	130.42	87.68	214.61	231.09	**0.90**	***0.04***	**1.32**	***3.25E−22***
Solyc11g045120	TIF SUI1	27.55	38.18	23.17	125.99	−0.05	1.00	**1.64**	***3.51E−24***
Solyc05g050200	TIF 1A	230.46	229.34	249.91	380.51	0.30	0.64	**0.65**	***1.56E−06***
Solyc06g082580	TIF 2 beta subunit-like	74.76	82.58	66.52	144.39	0.02	1.00	**0.73**	***0.033963***
Solyc05g054310	TIF 2 gamma subunit	7.79	4.81	6.59	12.18	−0.03	1.00	**1.26**	***7.34E−10***
Solyc12g099470	TIF 2 gamma subunit	31.65	29.71	32.73	55.40	0.25	0.80	**0.82**	***6.38E−08***
Solyc01g066690	TIF 2 gamma subunit	38.63	31.66	38.50	51.49	0.18	0.90	**0.62**	***3.74E−05***
Solyc11g072090	TIF e-2B gamma subunit	10.84	11.81	12.73	20.83	0.42	0.54	**0.74**	***7.91E−05***
Solyc08g081900	TIF-2	47.65	58.49	43.26	38.62	0.03	1.00	**−0.68**	***1.60E−06***
Solyc02g089070	TIF 3 subunit M	46.21	39.94	39.16	63.98	−0.04	1.00	**0.60**	***6.75E−05***
Solyc01g111720	TIF -3	22.31	28.73	39.06	48.83	**1.00**	***0.00***	**0.68**	***3.30E−06***
Solyc07g005830	TIF -3	25.22	18.58	30.01	30.04	0.44	0.41	**0.61**	***6.10E−04***
Solyc05g052690	TIF 3 subunit 5	12.51	7.22	10.31	12.25	−0.08	1.00	**0.68**	***0.003033***
Solyc10g079880	TIF 3 subunit 6	98.95	75.88	90.12	123.24	0.06	1.00	**0.62**	***1.00E−05***
Solyc02g078120	TIF 3 subunit 7	81.54	69.50	86.60	185.48	0.28	0.72	**1.34**	***1.63E−24***
Solyc07g042570	TIF 3 subunit 11	26.49	20.78	21.88	34.34	−0.08	1.00	**0.64**	***0.000307***
Solyc12g009960	TIF 4	268.11	369.80	148.96	261.71	**−0.65**	***0.04***	**−0.58**	**0.068**
Solyc02g091220	TIF 5	16.74	16.17	15.85	35.12	0.13	1.00	**1.04**	***6.67E−05***
Solyc03g115650	TIF 5A	608.59	468.38	380.09	134.97	−0.50	0.48	**−1.87**	***9.90E−47***
Solyc04g005510	TIF 5A	28.88	11.18	25.71	18.57	0.03	1.00	**0.66**	***0.006651***
Solyc07g053470	Translation EF A	22.40	30.45	26.24	48.64	0.45	0.62	**0.60**	***5.23E−05***
Solyc11g069700	EF 1-alpha	237.33	24.59	45.96	731.23	**−2.14**	***0.01***	**4.81**	***6.80E−03***
Solyc06g009970	EF 1-alpha	580.98	421.60	477.65	680.79	−0.08	0.98	**0.61**	***3.69E−06***
Solyc11g072190	EF beta-1	732.10	281.72	474.17	480.92	−0.43	0.31	**0.69**	***2.42E−07***
Solyc06g011280	EF 1-gamma	231.68	148.77	196.44	253.96	−0.04	1.00	**0.69**	***2.07E−07***
Solyc09g073000	EF Tu	31.89	40.16	51.87	85.99	0.90	0.06	**1.02**	***1.40E−14***
Solyc06g008940	EF Tu /EF1A	4.28	10.40	6.44	16.39	0.82	0.34	**0.58**	***0.003***
Solyc06g071790	EF Tu	111.53	144.68	109.46	99.93	0.16	0.94	**−0.61**	***1.00E−05***
Solyc01g098000	EF like	31.48	36.24	32.95	68.22	0.27	0.93	**0.83**	***3.13E−06***
Solyc04g009810	Pelota homolog probable translation F	8.86	5.97	7.27	14.56	−0.10	1.00	**1.20**	***0.003231***

The cutoff is 0.58 log2 fold change at 579HO B8 vs. 556AZ B8. The significant log2 fold changes are in bold font. The significant padj ≤ 0.05 are in bold and italics. * Average of three independent biological replicates. Green color cells show positive values and orange color cells show negative log2 fold change in 579HO as compared with 556AZ.

## Supplementary Materials

The following are available online at https://www.mdpi.com/2223-7747/9/12/1710/s1, Figure S1: Alignment of snoRNA cluster on Chr. 6 and Chr. 3. Figure S2: Alignment of snoRNA isolated from tomato fruit tissue, with the Arabidopsis and Rice box C/D snoRNA. Conserved external and internal C and D boxes and rRNA complementary sequence on snoRNA is marked (25S rRNA is target of identified box C/D snoRNA (on the basis of sequence alignment and homology)). Arabidopsis data reference, Lian Hu et al., Nucleic Acids Research 2001, 29:1623-1630. Figure S3: Quantitative RT-PCR analysis of rRNA and snoRNA genes in azygous (556AZ) and high polyamine accumulating homozygous (579HO) fruits during ripening, i.e., mature green (GR), breaker (BR), pink (PK) and red (RD). The bars represent standard errors of mean (N = 3). The different letters above bars indicate statistical significance at *p* < 0.05 as determined by Tukey’s test. Figure S4: Unedited Cytoscape gene expression network views: ribosomal related genes (A); all polymerase, ribosomal and translation related genes (B). Table S1: Summary of RNAseq analyses of differential expression of RNA polymerase genes in 556AZ and 579HO tomato fruit at breaker (B) and breaker+8 (B8) postharvest stages, Table S2: Summary of RNAseq analysis of differential expression of ribosomal protein genes in 556AZ and 579HO tomato lines at breaker (B) and breaker+8 (B8) postharvest stages. Table S3: Summary of RNAseq analyses of differential expression of RNA translation genes in 556AZ and 579HO tomato fruit at breaker (B) and breaker+8 (B8) postharvest stages. Table S4: Pearson correlation coefficients among the RNA polymerases genes. Table S5: Pearson correlation coefficients among the ribosomal genes. Table S6: Pearson correlation coefficients among the translation genes, Table S7: Pearson correlation coefficients among the polymerase, ribosomal, and translation related protein genes. Table S8: Primers used for Q-PCR and Northern blot.

## References

[1] Dupuis-Sandoval F., Poirier M., Scott M.S. (2015). The emerging landscape of small nucleolar RNAs in cell biology. Wiley Interdiscip. Rev. RNA.

[2] Leader D.J., Sanders J.F., Waugh R., Shaw P., Brown J.W. (1994). Molecular characterisation of plant U14 small nucleolar RNA genes: Closely linked genes are transcribed as polycistronic U14 transcripts. Nucleic Acids Res..

[3] Leader D.J., Clark G.P., Watters J., Beven A.F., Shaw P.J., Brown J.W.S. (1999). Splicing-independent processing of plant box C/D and box H/ACA small nucleolar RNAs. Plant. Mol. Biol..

[4] Ojha S., Malla S., Lyons S.M. (2020). snoRNPs: Functions in ribosome biogenesis. Biomolecules.

[5] Weis B.L., Kovacevic J., Missbach S., Schleiff E. (2015). Plant-specific features of ribosome biogenesis. Trends in Plant. Sci..

[6] Solymosy F., Pollàk T. (1993). Uridylate-rich small nuclear RNAs (UsnRNAs), their genes and pseudogenes, and UsnRNPs in plants: Structure and function. A comparative approach. Crit. Rev. Plant. Sci..

[7] Brown J.W.S., Shaw P.J. (1998). Small nucleolar RNAs and pre-RNA processing in plants. Plant. Cell.

[8] Kiss T., Marshallsay C., Filipowicz W. (1991). Alteration of the RNA polymerase specificity of U3 snRNA genes during evolution and in vitro. Cell.

[9] Taft R.J., Glazov E.A., Lassmann T., Hayashizaki Y., Carninci P., Mattick J.S. (2009). Small RNAs derived from snoRNAs. RNA.

[10] Zheng J., Zeng E., Du Y., He C., Hu Y., Jiao Z., Wang K., Li W., Ludens M., Fu J. (2019). Temporal Small RNA Expression Profiling under Drought Reveals a Potential Regulatory Role of Small Nucleolar RNAs in the Drought Responses of Maize. Plant. Genome.

[11] Goyal R.K., Kumar V., Shukla V., Mattoo R., Liu Y., Chung S.H., Giovannoni J.J., Mattoo A.K. (2012). Features of a unique intronless cluster of class I small heat shock protein genes in tandem with box C/D snoRNA genes on chromosome 6 in tomato (*Solanum lycopersicum*). Planta.

[12] Zhua P., Wanga Y., Qina N., Wanga F., Wanga J., Denda W., Zhua D. (2016). Arabidopsis small nucleolar RNA monitors the efficient pre-rRNA processing during ribosome biogenesis. Proc. Nat. Acad. Sci. USA.

[13] Streit D., Shanmugam T., Garbelyanski A., Simm S., Schleiff E. (2020). The existence and localization of nuclear snoRNAs in *Arabidopsis thaliana* revisited. Plants.

[14] Ku L.K., Romani R.J. (1970). The ribosomes of pear fruit. Plant. Physiol..

[15] Rattapanone N., Grierson D., Stein M. (1977). Ribonucleic acid metabolism during the development and ripening of tomato fruits. Phytochemistry.

[16] Speirs J., Brady C.J., Grierson D., Lee E. (1984). Changes in ribosome organization and messenger RNA abundance in ripening tomato fruits. Aust. J. Plant. Physiol..

[17] Ishiguro K., Arai T., Suzuki T. (2019). Depletion of S-adenosylmethionine impacts on ribosome biogenesis through hypomodification of a single rRNA methylation. Nucleic Acids Res..

[18] Lightfoot H.L., Hall J. (2014). Endogenous polyamine function--the RNA perspective. Nucleic Acids Res..

[19] Mattoo A.K., Handa A.K. (2008). Higher polyamines restore and enhance metabolic memory in ripening fruit. Plant. Sci..

[20] Handa A.K., Fatima T., Mattoo A.K. (2018). Polyamines: Biomolecules with diverse functions in plant and human health and disease. Front. Chem..

[21] Mehta R.A., Cassol T., Li N., Ali N., Handa A.K., Mattoo A.K. (2002). Engineered polyamine accumulation in tomato enhances phytonutrient content, juice quality and vine life. Nat. Biotechnol..

[22] Belda-Palazón B., Ruiz L., Martí E., Tárraga S., Tiburcio A.F., Culiáñez F., Farras R., Carrasco P., Ferrando A. (2012). Aminopropyltransferases involved in polyamine biosynthesis localize preferentially in the nucleus of plant cells. PLoS ONE.

[23] Upadhyay R.K., Fatima T., Handa A.K., Mattoo A.K. (2020). Polyamines and their biosynthesis/catabolism genes are differentially modulated in response to heat versus cold stress in tomato leaves (*Solanum lycopersicum* L.). Cells.

[24] Kramer G., Boehringer D., Ban N., Bukau B. (2009). The ribosome as a platform for co-translational processing, folding and targeting of newly synthesized proteins. Nat. Struct. Mol. Biol..

[25] Duc K., Batra S.S., Bhattacharya N., Cate J.H.D., Song Y.S. (2019). Differences in the path to exit the ribosome across the three domains of life. Nucleic Acids Res..

[26] Mattoo A.K., Chung A.H., Goyal R.K., Fatima T., Solomos T., Srivastava A., Handa A.K. (2007). Overaccumulation of higher polyamines in ripening transgenic tomato fruit revives metabolic memory, upregulates anabolism-related genes, and positively impacts nutritional quality. J. AOAC Internat..

[27] Srivastava A., Chung S.H., Fatima T., Datsenka T., Handa A.K., Mattoo A.K. (2007). Polyamines as anabolic growth regulators revealed by transcriptome analysis and metabolic profiles of tomato fruits engineered to accumulate spermidine and spermine. Plant. Biotechnol..

[28] Fatima T., Sobolev A.P., Teasdale J.R., Kramer M., Bunce J., Handa A.K., Mattoo A.K. (2016). Fruit metabolic networks in engineered and non-engineered tomato genotypes reveal fluidity in a hormone and agroecosystem specific manner. Metabolomics.

[29] McMahon M, Contreras A, Ruggero, D (2015). Small RNAs with big implications: New insights into H/ACA snoRNA function and their role in human disease. Wiley Interdiscip. Rev. RNA..

[30] Liang J., Wen J., Huang Z., Chen X.-P., Zhang B.-X., Chu L. (2019). Small nucleolar RNAs: Insight into their function in cancer. Front. Oncol..

[31] Brown J.W., Echeverria M., Qu L.H. (2003). Plant snoRNAs: Functional evolution and new modes of gene expression. Trends Plant. Sci..

[32] Chen C.L., Liang D., Zhou H., Zhou M., Chen Y.Q., Qu L.H. (2003). The high diversity of snoRNAs in plants: Identification and comparative study of 120 snoRNA genes from *Oryza Sativa*. Nucleic Acids Res..

[33] Kim S.H., Spensley M., Choi S.K., Calixto C.P.G., Pendle A.F., Koroleva O., Shaw P.J., Brown J.W.S. (2010). Plant U13 orthologues and oRPhan snoRNAs identified by RNomics of RNA from Arabidopsis nucleoli. Nucleic Acid Res..

[34] Janin M., Coll-SanMartin L., Esteller M. (2020). Disruption of the RNA modifications that target the ribosome translation machinery in human cancer. Molec. Cancer.

[35] Barsan C., Sanchez-Bel P., Rombaldi C., Egea I., Rossignol M., Kuntz M., Zouine M., Latché A., Bouzayen M., Pech J.-C. (2010). Characteristics of the tomato chromoplast revealed by proteomic analysis. J. Exp. Bot..

[36] Barsan C., Zouine M., Maza E., Bian W., Egea I., Rossignol M., Bouyssie D., Pichereaux C., Purgatto E., Bouzayen M. (2012). Proteomic analysis of chloroplast-to-chromoplast transition in tomato reveals metabolic shifts coupled with disrupted thylakoid biogenesis machinery and elevated energy-production components. Plant. Physiol..

[37] Pokalsky A.R., Hiatt W.R., Ridge N., Rasmussen R., Houck C.M., Shewmaker C.K. (1989). Structure and expression of elongation factor 1 alpha in tomato. Nucleic Acids Res..

[38] Talapatra S., Wagner J., Thompson C. (2002). Elongation factor-1 alpha is a selective regulator of growth factor withdrawal and ER stress-induced apoptosis. Cell Death Differ..

[39] Raabe K., Honys D., Michailidis C. (2019). The role of eukaryotic initiation factor 3 in plant translation regulation. Plant. Physiol. Biochem..

[40] Wang T.W., Lu L., Wang D., Thompson J.E. (2001). Isolation and characterization of senescence-induced cDNAs encoding deoxyhypusine synthase and eukaryotic translation initiation factor 5A from tomato. J. Biol. Chem..

[41] Bokszczanin K.L., Krezdorn N., Fragkostefanakis S., Muller S., Rycak L., Chen Y., Hoffmeier K., Kreutz J., Paupiere M.J., Chaturvedi P. (2015). Identification of novel small ncRNAs in pollen of tomato. BMC Genom..

[42] Trapnell C., Williams B.A., Pertea G., Mortazavi A., Kwan G., Van Baren M.J., Salzberg S.L., Wold B.J., Pachter L. (2010). Transcript assembly and quantification by RNA-Seq reveals unannotated transcripts and isoform switching during cell differentiation. Nat. Biotechnol..

[43] Anders S., Huber W. (2010). Differential expression analysis for sequence count data. Genome Biol..

[44] Benjamini Y., Hochberg Y. (1995). Controlling the false discovery rate. A practical and powerful approach to multiple testing. J. Roy. Stat. Soc. B Met..

[45] Shannon P., Markiel A., Ozier O., Baliga N.S., Wang J.T., Ramage D., Amin N., Schwikowski B., Ideker T. (2003). Cytoscape: A software environment for integrated models of biomolecular interaction networks. Genome Res..

