# Supplementary material for: Engineered Ripening-Specific Accumulation of Polyamines Spermidine and Spermine in Tomato Fruit Upregulates Clustered C/D Box snoRNA Gene Transcripts in Concert with Ribosomal RNA Biogenesis in the Red Ripe Fruit
Source: Plants (Basel). 2020 Dec 4;9(12):1710. doi: 10.3390/plants9121710 (PMC7762058; doi:10.3390/plants9121710)
Supplement: Supplementary file 1 [file plants-09-01710-s001.zip › plants-949738 - supplementary for XML.pdf]

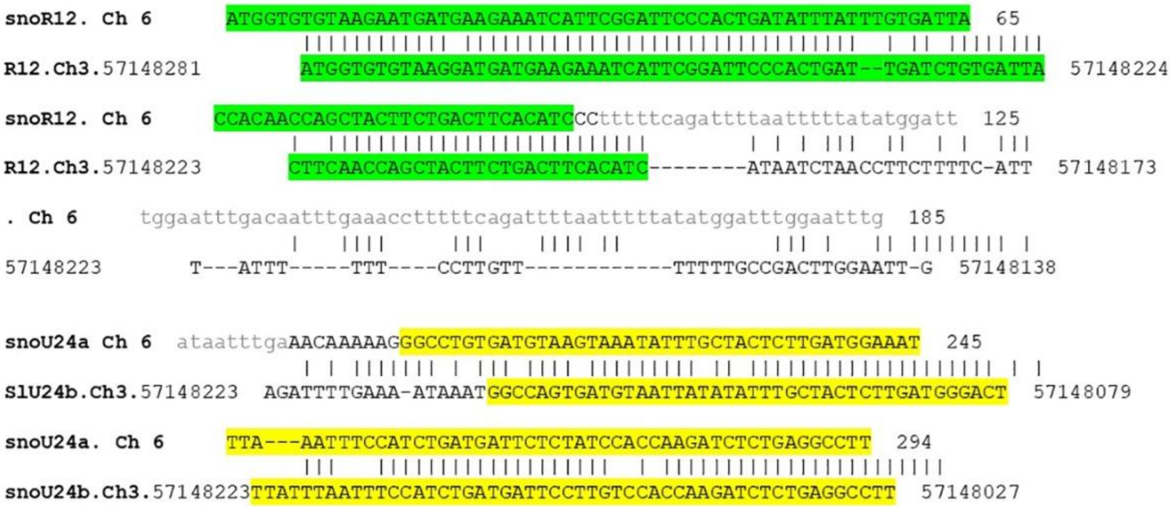

Figure S1. Alignment of snoRNA cluster on Chr. 6 and Chr. 3.

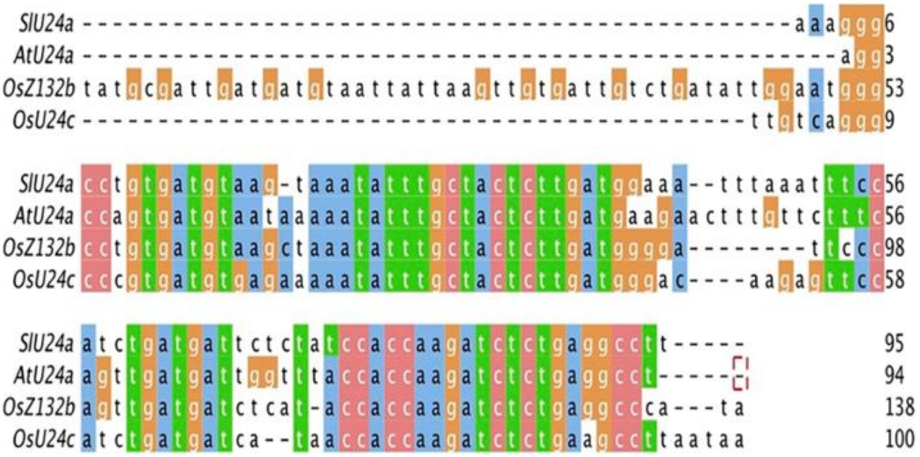

Figure S2. Alignment of snoRNA isolated from tomato fruit tissue, with the Arbidopsis and Rice box C/D snoRNA. Conserved external and internal C&D boxes and rRNA complementary sequence on snoRNA are marked. (25S rRNA is target of identified box C/D snoRNA (based on sequence alignment and homology). Arabidopsis data - Ref: Lian Hu et al., Nucleic Acids Research 2001; 29:1623-1630.

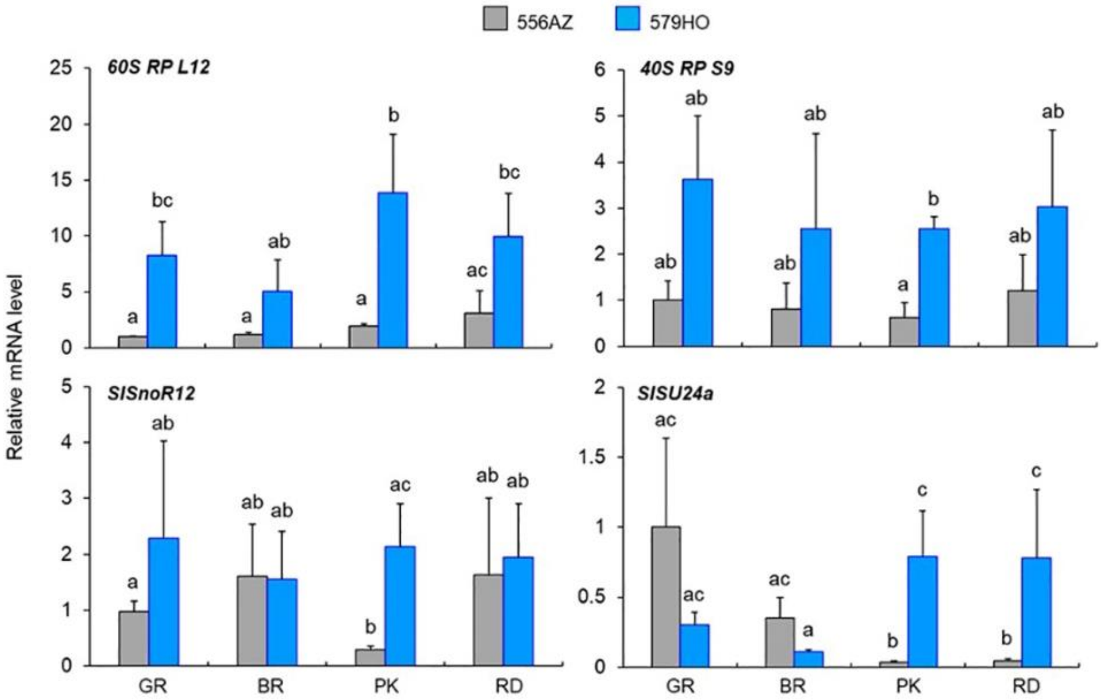

Figure S3. Quantitative RT-PCR analysis of rRNA and snoRNA genes in azygous (556AZ) and high polyamine accumulating homozygous (579HO) fruits during ripening, i.e., mature green (GR), breaker (BR), pink (PK) and red (RD). The bars represent standard errors of mean (N=3). The different letters above bars indicate statistical significance at $p < 0.05$ as determined by Tukey's test.

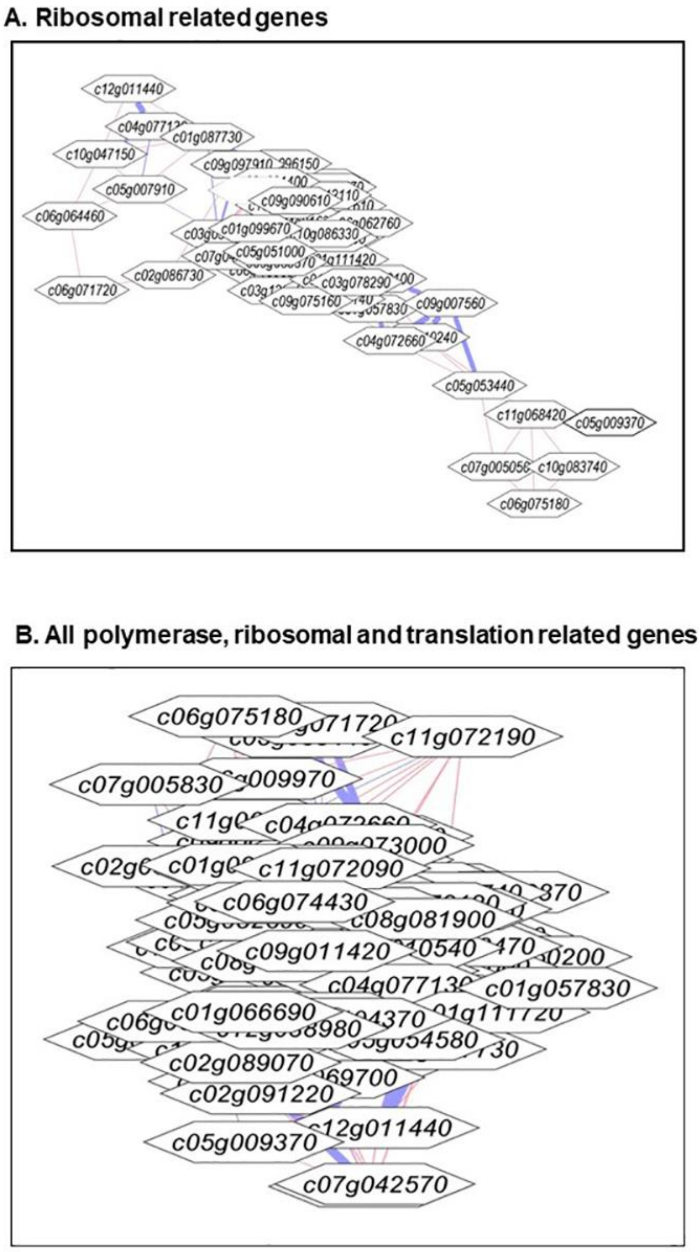

Figure S4. Unedited Cytoscape gene expression network views: ribosomal related genes (**A**); all polymerase, ribosomal and translation related genes (**B**).

Table S8. Primers used for Q-PCR and Northern Blot.

Primer	Sequence (5′ to 3′)	Experiment
qRTActin-Fwd	TGGTCGTACCACCGGTATTGTG	Q-PCR
qRTActin-Rev	AATGGCATGTGGAAGGGCATAC	Q-PCR
SlsnoR12-Fwd	TGATGGTGTGTAAGAATGAT	Q-PCR
SlsnoR12-Rev	AGTCAGAAGTAGCTGGTTGT	Q-PCR
qRTU6-Fwd	CGA TAC AGA GAA GAT TAG CAT G	Q-PCR
qRTU6-Rev	AAG CTC CAT TGT CAA ATT TT	Q-PCR
SlU24a-Fwd	AGGGCCTGTGATGTAAGTAA	Northern, Q-PCR
SlU24a-Rev	AGAACAAGGCCTCAGAGATC	Northern, Q-PCR
RTZ132-Fwd	AAAGGGCCTGTGATGTAAG	Northern
RTZ132-Rev	GGCCTCAGAGATCTTGGTGG	Northern
